# Supplementary material for: Evidence accumulation, not ‘self-control’, explains dorsolateral prefrontal activation during normative choice
Source: eLife. 2022 Sep 8;11:e65661. doi: 10.7554/eLife.65661 (PMC9457682; doi:10.7554/eLife.65661)
Supplement: Supplementary file 1. — Estimated Model Parameters: All Datasets. Parameter values were estimated using a differential-evolution Markov chain Monte Carlo method developed by Holmes and Trueblood, 2018. Parameters beginning with w indicate weighting parameters applied to different attributes. Datasets 1 and 2: proposed payoff to self vs. the default, proposed payoff to other vs. the default, fairness [|$Self − $Other|], and a constant bias toward the proposal; Dataset 3: tastiness and healthiness vs. the default as well as a constant bias toward the displayed food. B: choice-defining threshold. ndt: non-decision time. A priori constraints on the parameters, determined based on previous work and on theoretical limits, restricted them to the range indicated. In Datasets 2 and 3, columns indicated by different subscripts (a–c) differ significantly from each other at p<0.05, corrected for multiple comparisons. * Average parameter values collapsing over three independently collected samples of participants (see Supplementary file 2 for details of each sample separately). [file elife-65661-supp1.docx]

## Table S1. Estimated Model Parameters: All Datasets

|  |  | Dataset 1  (Altruism) | Dataset 2  (Altruism) | | |
| --- | --- | --- | --- | --- | --- |
| Parameter | *A priori* constraints | Natural Response | Natural Response | Focus on Ethics | Focus on Partner |
| w*_Self_* | -.5 to +.5 | .0043±.0011 | .0085±.0036^a^ | .0071±.0051^a^ | .0038±.0065^c^ |
| w*_Other_* | -.5 to +.5 | .0011±.0018 | .0011±.0044^a^ | .0050±.0052^b^ | .0062±.0046^b^ |
| w*_Fairness_* | -.5 to +.5 | .0011±.0032 | .0063±.0061^a^ | .0112±.0063^b^ | .0078±.0065^a^ |
| w*_Taste_* | -.5 to +.5 | - | - | - | - |
| w*_Health_* | -.5 to +.5 | - | - | - | - |
| w*_Constant_* | -5 to 5 | .0083±.0771 | .0529±.0755^a^ | .05521±.0906^a^ | .0566±.0654^a^ |
| *B* | 0 to +1.0 | .1360±.0221 | .1227±.0217^a^ | .1399±.0404^b^ | .1342±.0276^b^ |
| *ndt* | 0 to +2.0s | 1.049±.1899 | .7798±.2514^a^ | .7487±.2441^a^ | .8110±.2621^a^ |
|  |  |  |  | Dataset 3*  (Food Choice) |  |
|  |  |  | Natural Response | Focus on  Taste | Focus on Health |
| w*_Taste_* | -.5 to +.5 |  | .0058±.0036^a^ | .0056±.0043^a^ | .0027±.0033^b^ |
| w*_Health_* | -.5 to +.5 |  | .0005±.0021^a^ | .0002±.0022^a^ | .0042±.0035^b^ |
| w*_Constant_* | -5 to 5 |  | -.0060±.0449^a^ | .0069±.0408^b^ | -.0222±.0377^c^ |
| *B* | 0 to +1.0 |  | .106±.0257^a^ | .111±.0317^a,b^ | .112±.0260^b^ |
| *ndt* | 0 to +2.0s |  | .706±.165^a^ | .706±.182^a^ | .725±.189^a^ |
