# Supplementary material for: Evidence accumulation, not ‘self-control’, explains dorsolateral prefrontal activation during normative choice
Source: eLife. 2022 Sep 8;11:e65661. doi: 10.7554/eLife.65661 (PMC9457682; doi:10.7554/eLife.65661)
Supplement: Supplementary file 2. — Estimated Model Parameters: Dataset 3, separately by Samples 3a, 3b, and 3c. Parameter values were estimated using a differential-evolution Markov chain Monte Carlo method developed by Holmes and Trueblood, 2018. Parameters beginning with w indicate weighting parameters applied to tastiness and healthiness vs. the default as well as a constant bias toward the displayed food. B: choice-defining threshold. ndt: non-decision time. A priori constraints on the parameters, determined based on previous work and on theoretical limits, restricted them to the range indicated. Columns indicated by different subscripts (a–c) differ significantly from each other at p < 0.05, corrected for multiple comparisons. [file elife-65661-supp2.docx]

## Table S2. Estimated Model Parameters: Dataset 3, separately by Sample 3a, 3b, and 3c

|  | Sample 3a (N = 36) | | |
| --- | --- | --- | --- |
| Param. | Natural Response | Focus on Taste | Focus on Health |
| w*_Taste_* | .0086±.0024^a^ | .0086±.0025^a^ | .0026±.0032^b^ |
| w*_Health_* | -.0002±.002^a^ | -.0007±.0016^a^ | .0064±.0035^b^ |
| w*_Constant_* | -.0051±.040^a^ | .014±.042^b^ | -.0139±.0399^a^ |
| *B* | .1162±.0338^a^ | .12±.0289^a^ | .124±.0293^a^ |
| *ndt* | .607±.118^a^ | .623±.105^a^ | .643±.153^a^ |
|  | Sample 3b (N = 33) | | |
| w*_Taste_* | .0065±.0029^a,b^ | .0074±.0025^a^ | .0048±.0032^b^ |
| w*_Health_* | .0013±.0024^a^ | .001±.0024^a^ | .0041±.0032^b^ |
| w*_Constant_* | -.0108±.0535^a,b^ | -.0027±.043^b^ | -.0314±.0369^a^ |
| *B* | .1104±.0223^a^ | .1202±.042^a^ | .1195±.0244^a^ |
| *ndt* | .623±.105^a^ | .658±.174^a^ | .643±.153^a^ |
|  | Sample 3c (N = 44) | | |
| w*_Taste_* | .0030±.0028^a^ | .0019±.0023^a^ | .0011±.0025^b^ |
| w*_Health_* | .0003±.0022^a^ | .0003±.0021^a^ | .0025±.0024^b^ |
| w*_Constant_* | -.0031±.042^a^ | .0082±.0371^b^ | -.0221±.0357^a^ |
| *B* | .0951±.0138^a^ | .0969±.0169^a^ | .0958±.0137^a^ |
| *ndt* | .817±.140^a^ | .809±.192^a^ | .826±.175^a^ |
