# Supplementary material for: Evidence accumulation, not ‘self-control’, explains dorsolateral prefrontal activation during normative choice
Source: eLife. 2022 Sep 8;11:e65661. doi: 10.7554/eLife.65661 (PMC9457682; doi:10.7554/eLife.65661)
Supplement: Supplementary file 3. — Neural correlates of the drift diffusion model (DDM) across datasets. Regions are reported at a voxel level of p < 0.001, uncorrected and a whole-brain FWE cluster-corrected level of p < 0.05, unless otherwise noted. * Distinct peak within larger cluster. † Significant at p < 0.005, uncorrected, reported for completeness. [file elife-65661-supp3.docx]

## Table S3. Neural correlates of the drift diffusion model (DDM) across datasets

| Region | | BA | Cluster Size | Z score | x | y | z |
| --- | --- | --- | --- | --- | --- | --- | --- |
| *Dataset 1 (GLM 1a)* | | | | | | | |
| R | Ventral Anterior Cingulate Cortex | 32 | 57 | 4.44 | 0 | 30 | -9 |
| R | Inferior Frontal Gyrus | 47 | 314 | 5.31 | 39 | 27 | -6 |
| L | Inferior Frontal Gyrus | 47 | 560 | 5.75 | -30 | 27 | -6 |
| L | Dorsolateral Prefrontal Cortex | 45/46 | * | 4.89 | -57 | 21 | 24 |
| L | Dorsal Anterior Cingulate | 6/8/32 | 908 | 5.33 | -6 | 24 | 48 |
| L | Middle Temporal Gyrus | 21 | 135 | 4.23 | -60 | -27 | -18 |
| R | Inferior Parietal Lobule | 40 | 456 | 6.91 | 54 | -66 | 36 |
| L | Inferior Parietal Lobule | 40 | 530 | 6.54 | -36 | -84 | 39 |
|  |  |  |  |  |  |  |  |
| *Dataset 2 (GLM 1b)* | | | | | | | |
| R | Medial Prefrontal Cortex | 6/8/9/32 | 1612 | 5.16 | 0 | 47 | -14 |
| R | Dorsal Anterior Cingulate |  | * | 3.99 | 0 | 38 | 34 |
| L | Inferior Frontal Gyrus | 45/47 | 406 | 5.19 | -45 | 32 | -8 |
| R | Inferior Frontal Gyrus | 47 | 304 | 5.71 | 42 | 23 | -14 |
| L | Dorsolateral Prefrontal Cortex | 45 | 9† | 2.76 | -57 | 20 | 22 |
| L | Posterior Cingulate Cortex | 31 | 184 | 5.07 | -6 | -40 | 34 |
| L | Inferior Parietal Cortex | 39 | 292 | 5.77 | -42 | -70 | 37 |
| R | Inferior Parietal Cortex | 39 | 121 | 5.00 | 42 | -73 | 34 |
|  |  |  |  |  |  |  |  |
| *Dataset 3 (GLM 1c)* | | | | | | | |
| L | Dorsomedial Prefrontal Cortex | 6/8/9/32 | 3342 | 6.40 | -24 | 2 | 55 |
| R | Dorsal Anterior Cingulate Cortex |  | * | 6.12 | 6 | 38 | 34 |
| L | Inferior Frontal Gyrus | 45 | 82 | 5.00 | -30 | 26 | -2 |
| L | Middle Frontal Gyrus | 9 | 326 |  | -27 | 32 | 40 |
| L | Dorsolateral Prefrontal Cortex | 45/46 | * | 4.54 | -54 | 20 | 19 |
| R | Middle Temporal Gyrus | 21 | 90 | 4.43 | 57 | -13 | -14 |
| L | Precuneus | 7 | 355 | 4.74 | -9 | -61 | 58 |
| R | Caudate |  | 260 | 5.56 | 9 | 5 | 7 |
