# Supplementary material for: Evidence accumulation, not ‘self-control’, explains dorsolateral prefrontal activation during normative choice
Source: eLife. 2022 Sep 8;11:e65661. doi: 10.7554/eLife.65661 (PMC9457682; doi:10.7554/eLife.65661)
Supplement: Supplementary file 4. — Neural correlates of normative vs. hedonistic choice across datasets. Regions are reported at a voxel-level threshold of p < 0.001, uncorrected, and a minimum volume of k = 10 voxels, unless otherwise noted. * Significant at p < 0.005, uncorrected and minimum volume of k=20 voxels, reported for completeness. ** Distinct peak within larger cluster, reported for completeness. [file elife-65661-supp4.docx]

## Table S4. Neural correlates of normative vs. hedonistic choice across datasets

| **Region** | | **BA** | **Cluster Size** | **Z score** | **x** | **y** | **z** |
| --- | --- | --- | --- | --- | --- | --- | --- |
|  |  |  |  |  |  |  |  |
| *Dataset 1, Generous vs. Selfish (GLM2a)* | | | | | | | |
| L | Dorsomedial Prefrontal Cortex | 9/32 | 86 | 4.03 | -3 | 33 | 36 |
| R | Dorsolateral Prefrontal Cortex | 44/45 | 24 | 3.87 | 54 | 12 | 21 |
| L | Dorsolateral Prefrontal Cortex | 45/46 | 18* | 3.06 | -45 | 12 | 18 |
| R | Inferior Frontal Gyrus | 47 | 23 | 4.12 | 30 | 21 | -12 |
| L | Inferior Frontal Gyrus | 47 | 13 | 3.73 | -42 | 39 | -3 |
| L | Inferior Parietal Lobule | 40 | 14 | 3.56 | -60 | -54 | 39 |
|  |  |  |  |  |  |  |  |
| *Dataset 2, Generous vs. Selfish, Natural Focus trials only (GLM2b)* | | | | | | | |
| L | Frontal Pole | 10 | 11 | 3.38 | -9 | 62 | 13 |
| R | Dorsolateral Prefrontal Cortex | 46 | 38 | 3.68 | 57 | 23 | 25 |
| L | Inferior Frontal Gyrus | 47 | 381 | 5.21 | -42 | 20 | -8 |
| R | Inferior Frontal Gyrus | 47 | 10 | 3.47 | 33 | 17 | -11 |
| L | Dorsomedial Prefrontal Cortex | 9/32/24 | 2225 | 5.21 | -3 | 11 | 67 |
|  | Dorsomedial Prefrontal Cortex |  | ** | 4.79 | -9 | 38 | 37 |
|  | Dorsolateral Prefrontal Cortex |  | ** | 3.85 | -42 | 14 | 31 |
| L | Mid-Cingulate Cortex | 24 | 30 | 4.33 | -3 | -4 | 31 |
| R | Inferior Parietal Lobule | 40 | 20 | 3.66 | 48 | -37 | 46 |
| L | Inferior Parietal Lobule | 40 | 18 | 3.59 | -57 | -37 | 46 |
| R | Posterior Cingulate Cortex | 31 | 54 | 3.79 | 12 | -40 | 31 |
| R | Cerebellum |  | 21 | 3.57 | 0 | -52 | -23 |
| R | Inferior Parietal Lobule | 40 | 32 | 3.76 | 48 | -58 | 46 |
| L | Inferior Parietal Lobule | 40 | 180 | 4.42 | -39 | -67 | 46 |
| L | Lingual Gyrus | 18 | 37 | 3.64 | -9 | -73 | 1 |
|  |  |  |  |  |  |  |  |
| *Dataset 2, Generous vs. Selfish, Ethics Focus trials only (GLM2b)* | | | | | | | |
|  | *No regions significant* |  |  |  |  |  |  |
|  |  |  |  |  |  |  |  |
| *Dataset 2, Generous vs. Selfish, Partner Focus trials only (GLM2b)* | | | | | | | |
| L | Dorsomedial Prefrontal Cortex | 24 | 54 | -3.64 | -3 | 41 | 22 |
| R | Dorsolateral Prefrontal Cortex | 46 | 16* | -3.81 | 57 | 29 | 22 |
| R | Inferior Frontal Gyrus | 47 | 47* | -3.36 | 36 | 23 | -2 |
|  |  |  |  |  |  |  |  |
| *Dataset 3, Healthy vs. Unhealthy, Natural Focus conflict trials only (GLM2c)* | | | | | | | |
| L | Dorsomedial Prefrontal Cortex | 9 | 20* | 3.82 | -6 | 32 | 37 |
| L | Dorsolateral Prefrontal Cortex | 46 | 23* | 3.02 | -45 | 26 | 13 |
| L | Inferior Frontal Gyrus | 47 | 43* | 3.09 | -27 | 20 | -8 |
| R | Inferior Frontal Gyrus | 47 | 17 | 4.47 | 30 | 20 | -5 |
|  |  |  |  |  |  |  |  |
| *Dataset 3, Healthy vs. Unhealthy, Taste Focus conflict trials only (GLM2c)* | | | | | | | |
|  | *No regions significant* |  |  |  |  |  |  |
|  |  |  |  |  |  |  |  |
| *Dataset 3, Healthy vs. Unhealthy, Health Focus conflict trials only (GLM2c)* | | | | | | | |
| R | Anterior Cingulate Cortex | 24 | 15 | -3.94 | 6 | 29 | 10 |
|  |  |  |  |  |  |  |  |
