# Supplementary material for: Evidence accumulation, not ‘self-control’, explains dorsolateral prefrontal activation during normative choice
Source: eLife. 2022 Sep 8;11:e65661. doi: 10.7554/eLife.65661 (PMC9457682; doi:10.7554/eLife.65661)
Supplement: Transparent reporting form [file elife-65661-transrepform1.pdf]

Westbrook Centre
Milton Road
Cambridge CB4 1YG, UK
P 01223 855340
W elifesciences.org
T @elife

eLife's transparent reporting form

We encourage authors to provide detailed information *within their submission* to facilitate the interpretation and replication of experiments. Authors can upload supporting documentation to indicate the use of appropriate reporting guidelines for health-related research (see EQUATOR Network), life science research (see the BioSharing Information Resource), or the ARRIVE guidelines for reporting work involving animal research. Where applicable, authors should refer to any relevant reporting standards documents in this form.

If you have any questions, please consult our Journal Policies and/or contact us: editorial@elifesciences.org.

Sample-size estimation

- You should state whether an appropriate sample size was computed when the study was being designed
- You should state the statistical method of sample size computation and any required assumptions
- If no explicit power analysis was used, you should describe how you decided what sample (replicate) size (number) to use

Please outline where this information can be found within the submission (e.g., sections or figure legends), or explain why this information doesn't apply to your submission:

This project consists of new analyses of previously published functional neuroimaging datasets. The sample size of those datasets was determined by the maximum number of participants that could be run consistent with the budget (Datasets 1, 2, 3b, and 3c), or the total number of participants from Dataset 2 who could be re-contacted and agreed to participate in Dataset 3a. This information can be found in the Participants section of the manuscript.

Replicates

- You should report how often each experiment was performed
- You should include a definition of biological versus technical replication
- The data obtained should be provided and sufficient information should be provided to indicate the number of independent biological and/or technical replicates
- If you encountered any outliers, you should describe how these were handled
- Criteria for exclusion/inclusion of data should be clearly stated
- High-throughput sequence data should be uploaded before submission, with a private link for reviewers provided (these are available from both GEO and ArrayExpress)

Please outline where this information can be found within the submission (e.g., sections or figure legends), or explain why this information doesn't apply to your submission:

eLife Sciences Publications, Ltd is a limited liability non-profit non-stock corporation incorporated in the State of Delaware, USA, with company number 5030732, and is registered in the UK with company number FC030576 and branch number BR015634 at the address Westbrook Centre, Milton Road, Cambridge CB4 1YG.

Each experiment was performed only once. However, each experiment replicates key aspects of the other. For example, Datasets 1 and 2 both examine altruistic decision making, while Datasets 2 and 3 both examine the influence of attentional focus on choice. The same computational and neuroimaging analyses are applied to all three datasets, allowing for replicability of the key findings to be assessed, and replication of key results confirmed across all three studies. The results of these replications can be seen in the Results section of the manuscript, as well as Figure 4 in the main text, and Figures S2, S4, and S6-8 of the supplementary results section.

Participants were excluded if they had excessive head motion or technical difficulties during scanning. Details are reported in the Participants section of the Methods.

eLife Sciences Publications, Ltd is a limited liability non-profit non-stock corporation incorporated in the State of Delaware, USA, with company number 5030732, and is registered in the UK with company number FC030576 and branch number [illegible]

Statistical reporting

- Statistical analysis methods should be described and justified
- Raw data should be presented in figures whenever informative to do so (typically when N per group is less than 10)
- For each experiment, you should identify the statistical tests used, exact values of N, definitions of center, methods of multiple test correction, and dispersion and precision measures (e.g., mean, median, SD, SEM, confidence intervals; and, for the major substantive results, a measure of effect size (e.g., Pearson's r, Cohen's d)
- Report exact p-values wherever possible alongside the summary statistics and 95% confidence intervals. These should be reported for all key questions and not only when the p-value is less than 0.05.

Please outline where this information can be found within the submission (e.g., sections or figure legends), or explain why this information doesn't apply to your submission:

All statistical analyses and statistical results are reported in the Results and Supplementary Results sections of the manuscript, and in Figure 4 of the main manuscript as well as Figures S1-S8, and Tables S1-S4. Statistical tests used are reported, along with N and degrees of freedom. Where appropriate, means and standard deviations are reported, along with exact P-values and measures of effect size (i.e., Cohen's d).

(For large datasets, or papers with a very large number of statistical tests, you may upload a single table file with tests, Ns, etc., with reference to sections in the manuscript.)

Group allocation

- Indicate how samples were allocated into experimental groups (in the case of clinical studies, please specify allocation to treatment method); if randomization was used, please also state if restricted randomization was applied
- Indicate if masking was used during group allocation, data collection and/or data analysis

Please outline where this information can be found within the submission (e.g., sections or figure legends), or explain why this information doesn't apply to your submission:

N/A. All tests were performed within-subjects. Thus, all subjects in a study completed trials in all experimental conditions.

Additional data files ("source data")

- We encourage you to upload relevant additional data files, such as numerical data that are represented as a graph in a figure, or as a summary table
- Where provided, these should be in the most useful format, and they can be uploaded as "Source data" files linked to a main figure or table
- Include model definition files including the full list of parameters used
- Include code used for data analysis (e.g., R, MatLab)
- Avoid stating that data files are "available upon request"

Please indicate the figures or tables for which source data files have been provided:

Analysis code, ROI masks, and behavioral data for all studies, as well as functional imaging data for Dataset 1, is available on the Open Science Framework at https://osf.io/eqvwd/. Functional imaging analysis data for Datasets 2 and 3a is available on the Open Science Framework at https://osf.io/wa4cs/. Functional imaging data for Dataset 3b is available at https://osf.io/3rne9/. Because it is part of a larger on-going study examining neural correlates of obesity, functional imaging data for Dataset 3c is not yet publicly available. However, all pre-processed ROI data and computational modeling data used for analysis and to create figures is available on the Open Science Framework at https://osf.io/eqvwd/.

eLife Sciences Publications, Ltd is a limited liability non-profit non-stock corporation incorporated in the State of Delaware, USA, with company number 5030732, and is registered in the UK with company number FC030576 and branch number [illegible]
